# High cost enhances cooperation through the interplay between evolution and self-organisation

**DOI:** 10.1186/s12862-016-0600-9

**Published:** 2016-02-01

**Authors:** Enrico Sandro Colizzi, Paulien Hogeweg

**Affiliations:** Theoretical Biology, Utrecht University, Padualaan 8, Utrecht, 3524ZL The Netherlands

**Keywords:** Evolution of cooperation, Spatial model, Viscous populations, Travelling waves, Multi-level evolution

## Abstract

**Background:**

Cooperation is ubiquitous in biological systems, yet its evolution is a long lasting evolutionary problem. A general and intuitive result from theoretical models of cooperative behaviour is that cooperation decreases when its costs are higher, because selfish individuals gain selective advantage.

**Results:**

Contrary to this intuition, we show that cooperation can increase with higher costs. We analyse a minimal model where individuals live on a lattice and evolve the degree of cooperation. We find that a feedback establishes between the evolutionary dynamics of public good production and the spatial self-organisation of the population. The evolutionary dynamics lead to the speciation of a cooperative and a selfish lineage. The ensuing spatial self-organisation automatically diversifies the selection pressure on the two lineages. This enables selfish individuals to successfully invade cooperators at the expenses of their autonomous replication, and cooperators to increase public good production while expanding in the empty space left behind by cheaters. We show that this emergent feedback leads to higher degrees of cooperation when costs are higher.

**Conclusions:**

An emergent feedback between evolution and self-organisation leads to high degrees of cooperation at high costs, under simple and general conditions. We propose this as a general explanation for the evolution of cooperative behaviours under seemingly prohibitive conditions.

**Electronic supplementary material:**

The online version of this article (doi:10.1186/s12862-016-0600-9) contains supplementary material, which is available to authorized users.

## Background

When cooperation is costly to the individual but its benefits are equally shared in a group, one would expect progressively more selfish behaviour to be selected. This indeed happens when interactions between cooperative and selfish individuals are random. Instead, cooperation can be selected in a population when the interactions among cooperative and selfish individuals are structured, be it genetically, spatially or socially [1–6]. Population structure favours cooperation when it allows for cooperators to be in contact with each other more frequently than with selfish individuals [7].

The result is a (locally or globally) stable equilibrium configuration in which cooperators persist indefinitely, and selfish individuals may co-exist. For instance, this can be due to spatial clustering of cooperators [8], or to an inherent structure of the interaction network [9]. Then, the conditions for selfish individuals invading and overtaking a group of cooperators represent the limit to the stability of the solutions found. In general, these conditions state that higher costs of cooperation increase the selective advantage of selfish individuals.

While spatial structure alone can favour cooperators due to population viscosity, a growing body of experimental and theoretical work indicates that self-organised spatial patterns may have profound and complex effects on cooperative interactions, due to emergent heterogeneities in the local distributions and densities of cooperators and selfish individuals [10–19]. For instance, as a population of cooperators invade empty space, its expansion front can be enriched in altruistic individuals, while selfish individuals lag behind [13, 15, 18]. Alternatively, mutualistic interactions that are favoured in a resident population can be automatically broken on such expansion front [14]. Furthermore, spatial self-organisation can sort cooperative strains from selfish ones [12, 20–24], thus limiting the spread of the latter.

The customary approach to study the stability of cooperation (under a specific set of assumptions) consists of fixing the strategies of the interacting cooperative and selfish individuals, and analyse the population dynamics of the system (e.g. whether cooperators and defectors coexist, or if one lineage outcompetes the other). Because such pre-determined strategies do not mutate over time, their evolutionary stability remains unexplored.

Although exceptions to this approach exist (e.g. [8, 22, 25, 26]), little is known about what strategies evolve (by mutation and selection) and how they feedback on the spatial self-organisation of a population, even though it is clear that spatial self-organisation affects the population dynamics of cooperative traits (see examples above). Here, we seek to study this feed-back between evolution and self-organisation with a minimal model where individuals can evolve the degree of cooperation in a spatially extended system.

We model the cooperative trait in terms of public good production (inspired by social dynamics in microbes [27, 28]), and we let the amount of public good produced mutate in a continuous fashion. Thus, we can study the long term evolutionary dynamics of cooperation without preconceiving the extent to which individuals cooperate or defect. As we will show, selfish individuals that produce zero public good evolve readily at higher costs, and quickly invade cooperators. Rather than leading to global extinction, this enables cooperators to thrive and selects for a higher degree of public good production over evolutionary time scales.

## Results

The model is a straightforward implementation of a population in which individuals replicate depending on the amount of public good produced in their close neighbourhood.

**Fig. 1:**
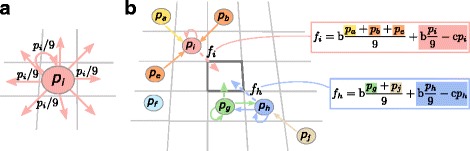
The model. **a** The world is a square lattice with connectivity *k*=8 (every node has 8 neighbours) and wrapped boundary conditions. Individuals produce public good at rate *p*, shared in equal parts (*p*/9) among all neighbouring nodes and self. **b** Individuals compete for reproduction into an adjacent empty spot. Probability of reproduction depends on fitness *f*_*i*_, which is the difference between benefits and costs. The sum of the public good an individual collects from itself and the neighbours, if any, is *p*_sum_=(*p*_*a*_+*p*_*b*_+…+*p*_*i*_)/9, and confers a benefit b·*p*_sum_. Individuals pay a fitness cost proportional to the public good they produce: c·*p*. Successful reproduction yields a copy of the selected individual. Mutations occur with probability *μ* and change the public good production rate by a small random number chosen uniformly in the interval [−*δ*/2,*δ*/2]. Individuals have a small probability *k*_move_ to move to a random adjacent node, and can die with probability *k*_death_, leaving the node empty (See Methods for the details of the models)

Individuals are embedded on a lattice. They may reproduce, die or move (locally). Competition for reproduction into neighbouring empty nodes is based on fitness, calculated as the difference between benefits and costs (Fig. 1). An individual benefits from the public good produced in its neighbourhood, but pays a cost for producing it. Thus, public good production is a cooperative trait. We assume that reproductive success is solely based on cooperation, so that individuals do not reproduce if public good in their neighbourhood is insufficient. Mutations slightly change the offspring’s production rate (see Methods for details).

### High cost leads to the evolution of larger public good production

We set the benefits per unit of public good b=10, and we let the spatial self-organisation and the evolutionary dynamics unfold under different costs c.

**Fig. 2:**
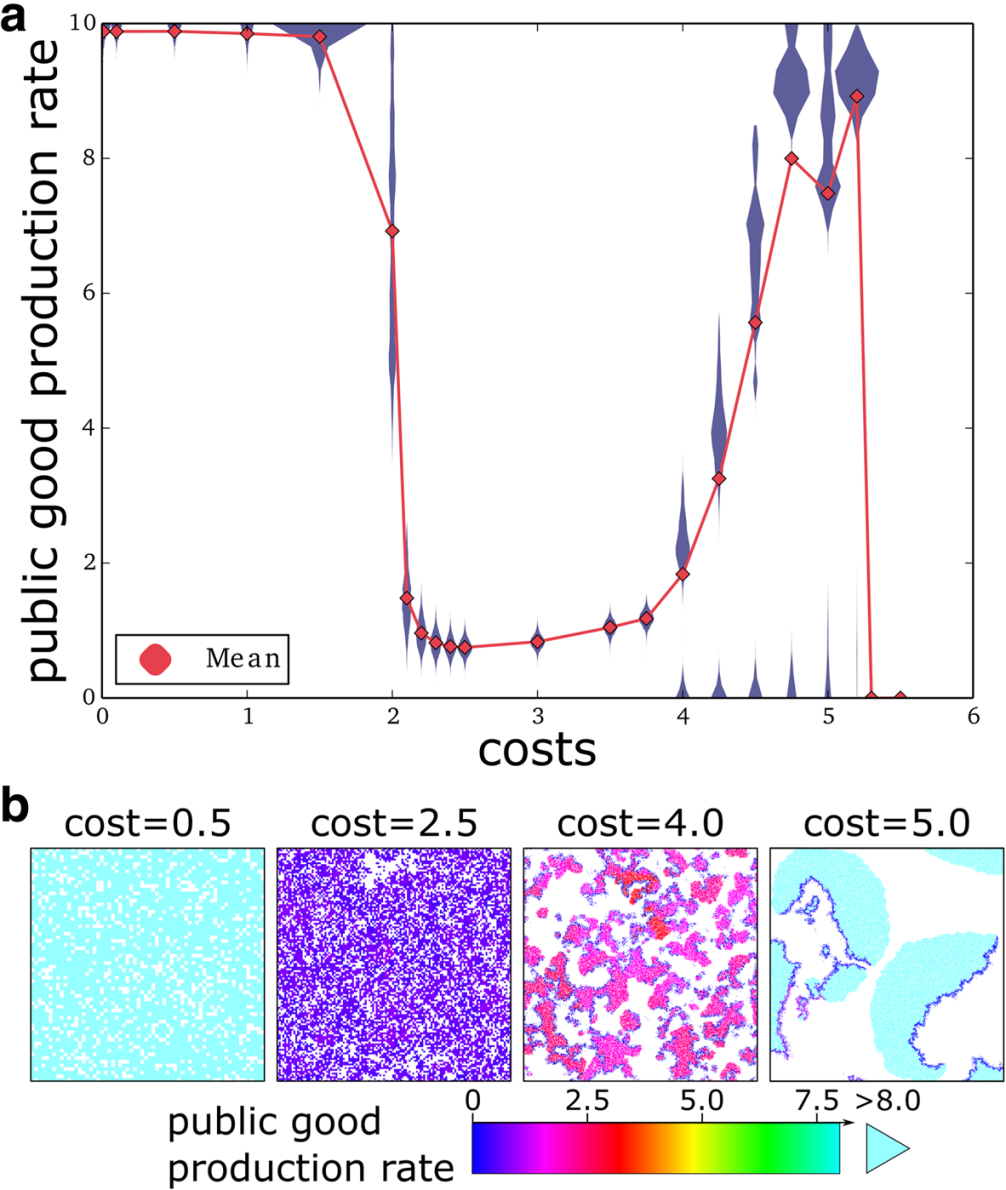
An increase in costs results in an evolutionary increase in cooperation. **a** Evolutionary steady state distribution (in blue) and mean (red diamond) of public good production are plotted for different values of costs (benefits are kept constant). Parameters: benefit (per unit of public good produced) b=10, *k*_death_=0.2, *k*_move_=0.02, *μ*=0.05, *δ*=0.1. The maximum public good production is set to *p*_max_=10. **b** Snapshots of the lattice at evolutionary steady state. Colour coding depends on public good production rate. White is background. Lattice dimensions used for the simulations from left to right: 256^2^, 512^2^, 2048^2^, 2048^2^ (1/16 of the lattice is displayed for clarity, see Additional file 1: Section 1 for the full snapshots)

When costs are much smaller than benefits (c≤1.5, Fig. 2), the public good production steadily increases because an individuals’ own production increases its fitness, rather than decreasing it. Moreover, because replication is a local process, mutants with higher than average production rates benefit from each other due to limited dispersal [7–9], outcompeting more selfish lineages. Thus, cooperation is maximised in the long run. For increasing costs, public good production suddenly drops (2≤c≤3, Fig. 2). In this regime, the clustering advantage of cooperators is insufficient and more selfish individuals replicate the most because, by producing less, they pay a lower cost. Eventually, public good production stabilises at the minimum value needed for survival.

Strikingly, further increasing costs leads to an increase in cooperation (c>3, Fig. 2, see Additional file 1: Section 1 for the full snapshots of the system). The distribution of public good production is bimodal at evolutionary steady state, with most of the population having higher rates of public good production and a minority producing almost no public good at all.

### The long term evolutionary dynamics of cooperation at high cost

**Fig. 3:**
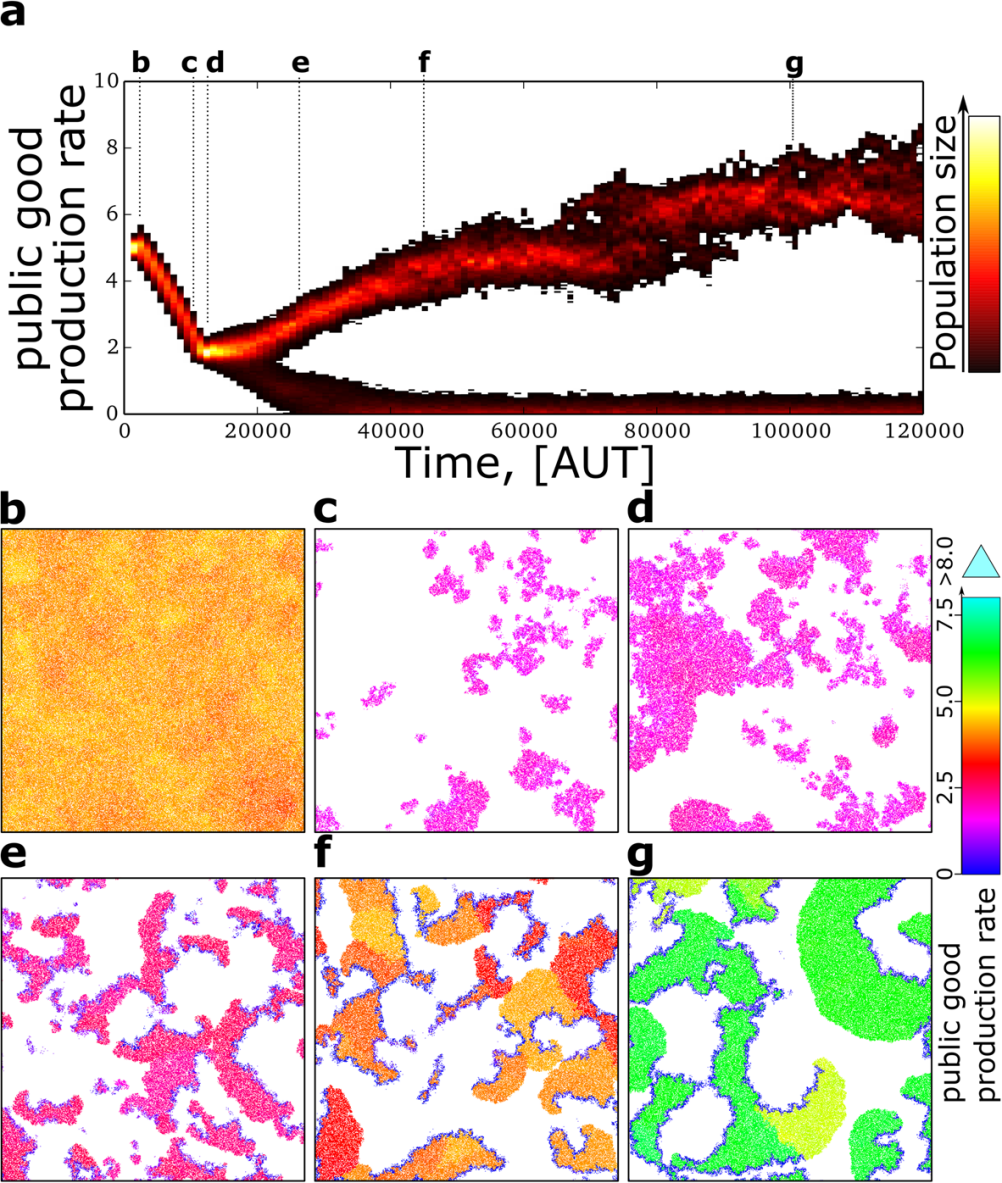
Evolutionary dynamics of public good production. **a** At each time point the distribution of public good production in the lattice is plotted as a heat map. **b**-**g** Snapshots of the lattice at subsequent time steps (letters correspond between time plot and snapshots). Costs (per unit of public good) c=4.5, other parameters and colour coding in the snapshot as in Fig. 2. Lattice size =2048^2^ (1/16 of the lattice is displayed for clarity). Time units are Monte Carlo steps. See also Additional file 1: Section 2 for the full snapshots, and movie at [29]

Following the evolutionary and the spatial dynamics of a single case elucidates why cooperation increases and persists for higher costs (c=4.5, Fig. 3, video at [29]). When we initialise the system with highly producing individuals, public good production decreases rapidly due to strong selection for selfishness (compare Fig. 3b and c). Where public good production drops below the minimum for survival, large patches of individuals go extinct (Fig. 3b). The surviving individuals can expand into the empty space (Fig. 3c). As the expansion progresses, a selfish and cooperative lineage separate from each other (Fig. 3d). The selfish strain evolves to zero public good production, becoming incapable of autonomous persistence and relying on the public good produced by cooperators for survival.

**Fig. 4:**
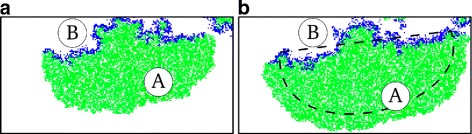
The movement of a travelling wave. Magnification (200 x 100) of the same portion of the lattice at 10 time steps distance. **a** the wave-front, composed of cooperative individuals (in green), **b** the back, composed of selfish individuals (in blue). The snapshots are from the same simulation run as in Fig. 3, at time steps 400,000 (left) and 400,010 (right). The dashed line in the right pane marks approximately the position of the wave in the left pane. Colizzi and Hogeweg [29] shows one such travelling wave in a video

While the two strains differentiate from each other, they organise spatially to form travelling waves (Fig. 3e and 3f, similar to [23, 24]). Cooperators constitute the front of a wave, and expand into empty space by replicating into it (generation after generation); selfish individuals invade those cooperators, and constitute the back of the wave. Selfish individuals leave empty space behind a wave after they die, causing the semblance of movement (See Fig. 4 and [29] for videos). The progression of a wave, however, happens on a time scale that is much longer than the life time of an individual, which in turn experiences a fairly constant environment throughout its life. Because waves persist longer than individuals, they can integrate information over several generations.

New waves are “born” from the collisions of older waves. As cooperators on the front evolve to larger public good production, waves become larger (compare Fig. 3e, f and g, and Additional file 1: Section 2 for the full snapshots). The formation of spatial patterns allows the populations in the system to persist indefinitely despite selfish individuals continuously invading cooperators (Fig. 3g and Additional file 1: Section 6), provided that the lattice is much larger than the spatial patterns (see Additional file 1: Section 3).

### Spatial population dynamics of cooperators and selfish individuals

Spatial self-organisation drives the evolution of cooperation in the system. When spatial patterns are destroyed, e.g. by mixing, only selfish individuals are selected and public good production decreases, leading to global extinction (Additional file 1: Section 4), in accordance with the result that random interactions favour selfish behaviour.

**Fig. 5:**
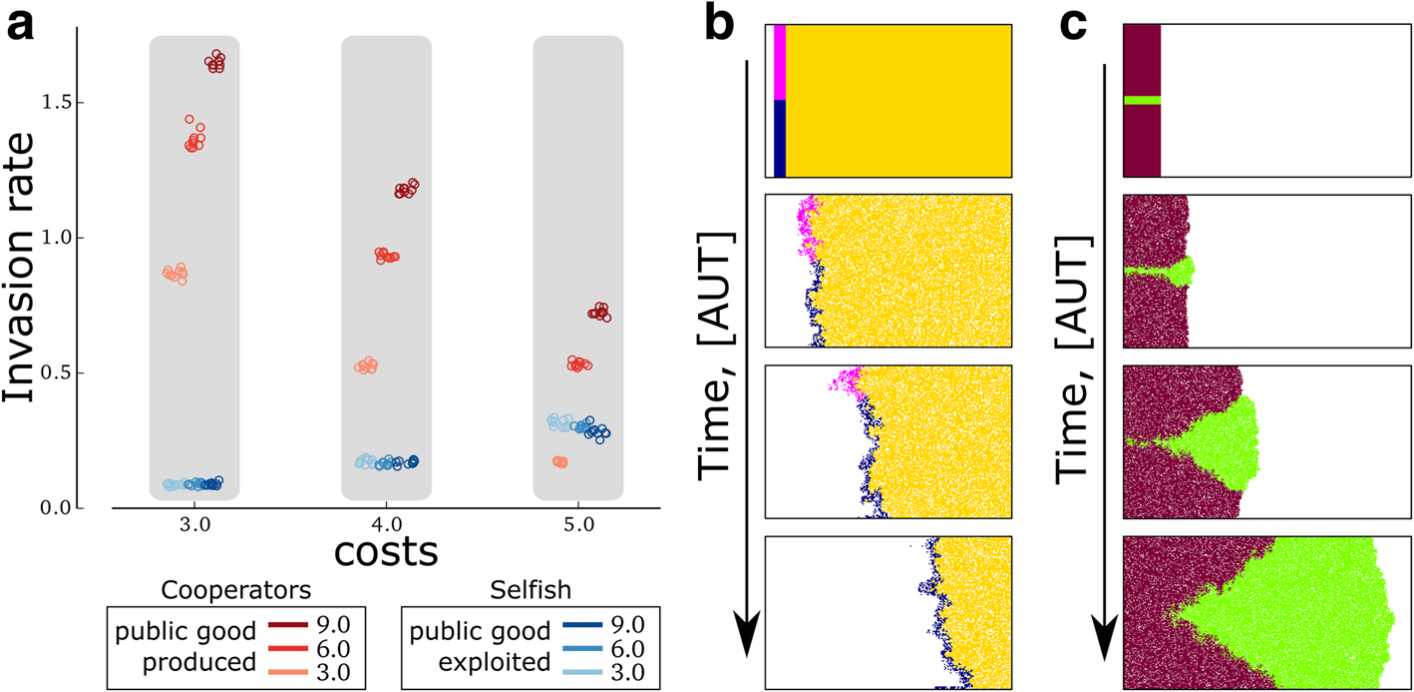
Invasion rates and spatial dynamics of competition for cooperative and selfish individuals. **a** Cooperators’ invasion rate increases with larger public good production and lower costs; selfish invasion rate increases with increasing costs and it is insensitive to the production rates of the cooperators that support them. The invasion rates for cooperators invading empty space (red circles) and selfish individuals invading a population of cooperators (blue circles) was measured for each combination of cost and public good production rate (benefit is constant, and set to b=10) in 10. **b** A population with lower public good production rate out-competes one with larger production in the back of a wave. Parameters: cooperators (yellow wave-front) *p*_yellow_=6.0, selfish individuals (magenta and blue wave-back) *p*_blue_=0.2 and *p*_magenta_=1.0. **c** The population with a larger production rate out-competes the one with lower production at the front of a wave. Parameters: *p*_purple_=5.0, *p*_green_=6.0. For both **a** and **b**, *μ*=0, b=10, c=4.0, background in white. Other parameters as in Fig. 2

To unravel the interplay between the two lineages and their spatial organisation, we analysed the spatial population dynamics for cooperative and selfish individuals separately. To this end, we shaped the lattice into a long, narrow strip, and set the mutation rate to zero (see Material and Methods for details). Cooperators expand faster into empty space when they produce more public good, and slower when costs are higher (Fig 5a, red). When two clustered populations compete at the expansion front (Fig 5c), the one with the largest public good production wins because, by replicating faster, it occupies space before the competing one and eventually overtakes the entire wave front.

The replication rate of selfish individuals invading a population of cooperators is higher when the cost of public good production is higher, and it is insensitive to how much public good is produced (Fig 5a, blue). Clearly, when two strains compete in the back of a wave, the winner is the more selfish one (Fig 5b).

The picture emerging from these experiments is that different selection pressures operate depending on the spatial context: a population expanding into empty space (the wave front) is selected for higher degrees of cooperation (in agreement with [12, 13, 15, 18]), competition in the back (behind the wave front) selects for more selfishness. Importantly, even though costs, benefits and fitness function are the same, spatial pattern formation automatically segregates these two opposing evolutionary pressures to spatially different contexts so that they do not balance each other: hence the evolution of a cooperative and a selfish lineage.

In the full system, cooperators and selfish individuals are ecologically and evolutionarily interdependent (Additional file 1: Section 5). The two lineages establish an evolutionary feedback mediated by their spatial organisation. Empty space is generated by selfish individuals after invading a population of cooperators. Therefore, the condition for increasing public good production, i.e. the availability of continuous empty space, is mediated by the invasion dynamics of the selfish lineage. With higher costs, selfish individuals propagate faster, and space is left empty at a higher rate. The larger the empty space, the more cooperators can increase public good production. This evolutionary feedback reaches an evolutionary steady state because highly producing cooperators reduce the empty space faster upon faster expansions (Fig 5a). We checked the long term stability of the steady state (Additional file 1: Section 6).

Altogether, cooperation evolves to a higher degree for higher costs due to an emergent feedback between self-organised interaction structures (the spatial patterns) and the evolution of the individuals composing them.

### Robustness to parameter change

Our results are robust when death rates, movement rates and benefits are changed, provided that benefits-to-costs ratio is maintained (Additional file 1: Section 7). At lower costs, individuals directly benefiting themselves with their own public good are sometimes dubbed *weak* altruists [7, 30] (in our case, for *c*<*b*/(*k*+1), with *k*+1=9 the connectivity of the lattice including self), whereas they are considered *strong* altruists when their public good is only shared among others (in the context of game theory, these situations are called, respectively, snowdrift game and prisoner’s dilemma [25]). In our spatial model, we observed no qualitative difference in the evolutionary dynamics when individuals did or did not benefit from their own public good (Additional file 1: Section 8). This could be expected because individuals’ own payoffs at high costs are negative in both models. Indeed, in both cases the evolving populations underwent speciation of a selfish and a cooperative lineage.

Weak or strong altruism do make a difference in the corresponding well-mixed systems, where strong altruists go extinct at lower costs, while weak altruists maximise public good production (Additional file 1: Section 9).

## Discussion

It is known that during population range expansion, cooperation can be promoted on the front of the expansion range [12, 13, 15, 18]. One could argue that in these models cooperation could evolve only as long as empty space is available, and should eventually be out-competed globally by selfish strategies when the invasion dynamics reach an end. Here we have shown that selfish individuals provide the empty space to allow continuous expansion within a limited area.

Generally, stable solutions to the problem of cooperation are based on the condition that at equilibrium selfish individuals do not locally invade a population of cooperators. For instance, it is well known that cooperation can be stabilised in spatially extended systems, as cooperators cluster and segregate selfish individuals to the edges of those clusters [8, 19, 31]. A side effect of these solutions is that higher costs undermines the stability of such clusters. In the parameter region where travelling waves do not form (Fig. 2, for c≤3) our work is in agreement with those results in that there exists an inverse relationship between costs and cooperation (e.g. [1, 2, 6]), and in particular with the heuristics [9] that cooperation evolves when the benefit-to-cost ratio is larger than the connectivity of the lattice (b/c>*k*, while it becomes progressively more unlikely when b/c approaches *k*).

However, we have shown that a novel class of solutions exists at high costs, where large degrees of cooperation are maintained in a locally out-of-equilibrium fashion, with selfish individuals always successfully invading cooperators and setting the stage for the evolutionary increase and the global stability of cooperation. We conclude that spatial self-organisation can reverse the relationship between costs and cooperation, thus extending the evolutionary viability of cooperation to higher costs.

Our results rest on two assumptions: population size can vary and some degree of cooperation is necessary for reproduction. Variable population size is obviously realistic, even though it is not often included in evolutionary models of cooperation. Although the assumption of necessary cooperation is not always met, it is reasonable in several cases. Examples in microbiology include, cooperative protection or cooperative virulence in bacterial infections [32, 33]; invertase production in yeasts while growing on sucrose [34]; siderophore production in iron-limited environments [35–37], cooperative secretion of digestive enzyme in microbial hunting [27, 38]. Outside the microbial world, situations where our model may apply are e.g. dangerous behaviours in cooperative nest defence [39], and replication *in trans* in prebiotic evolution [40, 41].

Two recent studies have come to conclusions that at first sight are similar to ours [17, 19]. By making the assumption, as we do, that the lack of cooperation leads to death, they observe (quasi) static spatial patterns in which cooperation is maintained because despite relatively high costs, clusters of cooperators cannot be invaded by selfish individuals.

In contrast, cheaters can always invade in our system, and do so faster when costs are higher. This shows that costs are qualitatively higher in our model. Cooperation is maintained despite, and due to the evolution of true cheaters (in the sense of [42]). Furthermore, we show that the amount of public good produced by an individual increases in evolutionary times, whereas the evolutionary stability of the solutions in [17, 19] is left unexplored, and only the long population dynamical transient is analysed.

More in general, the importance of spatial self-organisation for understanding the population dynamics of cooperators and defectors has been highlighted both from a theoretical [10] and from an experimental point of view [43]. Here we make a similar point, but with an evolutionary twist: in our case selfish individuals are not merely a burden to cooperators; instead, their emergence as a separate lineage is necessary for the evolution of high degrees of public good production because they generate the spatial conditions in which cooperators thrive and evolve.

Allowing mutations to change public good production in a continuous range resulted in the evolution of two separate strains, a selfish and a cooperative one. The evolution of stable heterogeneity in a population has been observed before in models of cooperation [22, 25, 44]. Here, besides stressing that the evolution of two lineages from a single ancestral one might be a rather general feature of models with variable investments (as very simple assumptions were needed, in contrast to [25]), we make the case that true cheating behaviour (*sensu* [42]) is actually functional and beneficial to the long term evolution of cooperation.

## Conclusions

In conclusion, besides extending the theoretical limits of cooperation, our results broaden the search image of cooperative behaviour in nature by suggesting that there need not be a strict trade-off between costs and benefits; rather, a wider view of the self-organised eco-evolutionary processes must be taken into account to understand the occurrence of costly cooperation.

## Methods

### General system

Our system is an individual-based, Monte Carlo simulation run on a square lattice with connectivity *k*=8 and toroidal boundary conditions. The nodes of the lattice can be empty or occupied by at most one individual. Individuals produce public good with rate *p* per time step (alternatively, *p* can be considered the degree of altruism of an individual). An individual *i* produces *p*_*i*_ public good per time step (0≤*p*_*i*_≤10), which is divided equally among neighbouring nodes and self, each receiving *p*_*i*_/9 public good. All *n* neighbours, in turn, share a fraction of the public good they produce *p*_{1,2,…,*n*}_/9 with individual *i*. The benefit from the public good received from each neighbour and from self is $B_{i} = \mathrm {b} \left (\frac {p_{i}}{9} + \frac {1}{9} \sum \limits _{j=1}^{n} p_{j} \right)$, where b is the benefit per unit of public good. Individuals pay a cost proportional to the public good they produce C_*i*_=c*p*_*i*_. Public good is not accumulated over multiple time steps. The fitness of an individual is the difference between benefits and costs: *f*_*i*_=B_*i*_−C_*i*_ (set to zero if costs exceed benefits).

Each Monte Carlo step, all nodes are updated in random order (although synchronous updating rules do not affect results). If a node is empty, the individuals in its neighbourhood (if any) compete for replication. Assume an individual *i* is competing with *m* other individuals, and let us name *f*_tot_ the sum of the fitness of all individuals competing for the same empty node, then $f_{\text {tot}} = f_{i} + \sum \limits _{k=1}^{m} f_{k}$. Individual *i* is chosen for reproduction over its competitors with probability $\mathrm {P}(i~\text {replicates}) = \frac {f_{i}}{ f_{\text {tot}}} \left (1 - e^{-f_{\text {tot}}} \right)$. The term in parenthesis is the probability that at least an individual replicates, which models the assumption replication should be more frequent in a neighbourhood where there is more public good, and conversely it should be rare if little public good is produced. Notice that this term does not affect death.

Upon successful replication, mutations may happen with probability *μ* and affect *p* by adding a small random number drawn with uniform probability from the interval [−*δ*/2,*δ*/2]. If a node is not empty, with probability *k*_move_ its content is swapped with that of a randomly chosen adjacent node. Moreover, every non empty node can turn to empty with probability *k*_death_. See Fig. 1 for a cartoon of the model and the caption of Fig. 2 for the actual values of the parameters. The algorithm is implemented using the CASH libraries [45].

### Invasion dynamics of cooperators and selfish individuals (Fig. 5)

We modified the system described above as follows: 1) we shaped the lattice into a narrow strip of arbitrary length; 2) we changed the boundary conditions to no-flux, and in particular we removed individuals when they moved or replicated into a boundary node of the lattice; 3) we set mutation rates to zero to better focus on spatial population dynamics. The rules for the local dynamics remained the same as above. We initialised all populations on one side of the lattice and waited until they reached the other side. For cooperators this meant that they invaded empty space, whereas selfish individuals invaded a resident homogeneous population of cooperators. In all cases, the number of Monte Carlo time steps it took for the first individuals to arrive to the other side of the lattice (generation by generation) was recorded. The invasion rate plotted in Fig. 5 was calculated as the length of the space invaded divided by the time it took for the population to invade it.

## Availability of data

The code used to run the simulations is available at http://bioinformatics.bio.uu.nl/enrico.

## Additional file

Additional file 1
**Contains the full snapshots of the lattice, as well as tests of parameters and structural robustness, as described in the main text.** (PDF 16179 kb)
