# Supplementary material for: High cost enhances cooperation through the interplay between evolution and self-organisation
Source: BMC Evol Biol. 2016 Feb 1;16:31. doi: 10.1186/s12862-016-0600-9 (PMC4736645; doi:10.1186/s12862-016-0600-9)
Supplement: Additional file 1 — Contains the full snapshots of the lattice, as well as tests of parameters and structural robustness, as described in the main text. (PDF 16179 kb) [file 12862_2016_600_MOESM1_ESM.pdf]

SUPPLEMENTARY MATERIAL
High cost enhances cooperation through the interplay between evolution and self-organisation

Enrico Sandro Colizzi, Paulien Hogeweg

January 20, 2016

1 Full snapshots of the system at evolutionary steady state for different costs

The snaphsots of the lattice at evolutionary steady state for different costs (main text, Fig. 2) are magnified to highlight the spatial patterns. The full snapshots are presented here in Fig. S1, S2 and S3 and the correct proportions between different lattices are maintained. In the parameter conditions where spatial patterns do not form, i.e. $c \leq 2.5$ a smaller lattice was used (because simulations are computationally expensive). A larger lattice was used for lerger costs (see Supplementary Section 3 for the consequences of smaller lattice with large spatial patterns).

2 Full snapshots of the evolutionary dynamics of public good production

The snaphsots of the lattice at evolutionary steady state for different costs (main text, Fig. 3) are magnified to highlight spatial pattern formation. The full snaphsots are presented here in Fig. S4, S5, S6, S7, S8, S9, S10.

3 Evolutionary consequences of small vs. large lattice

Because spatial pattern dynamics play an important role, the size of the lattice must be large enough to accommodate them. When costs are high relative to benefits, the spatial scale of pattern formation may become comparable to the lattice size. When this is the case, extinction is often observed as a consequence of wave-wave collision (see snapshots in Fig. S11), or because the front of a wave touches its own back (Fig. S12, in both cases $c = 5.3$, recall that lattice has wrapped boundary conditions). Hence, results in the main text may be extended to higher costs if lattice size were larger (at the expense of computational load). However, the evolutionary mechanism by which cooperation increases in the system remains the same as that reported in the main text.

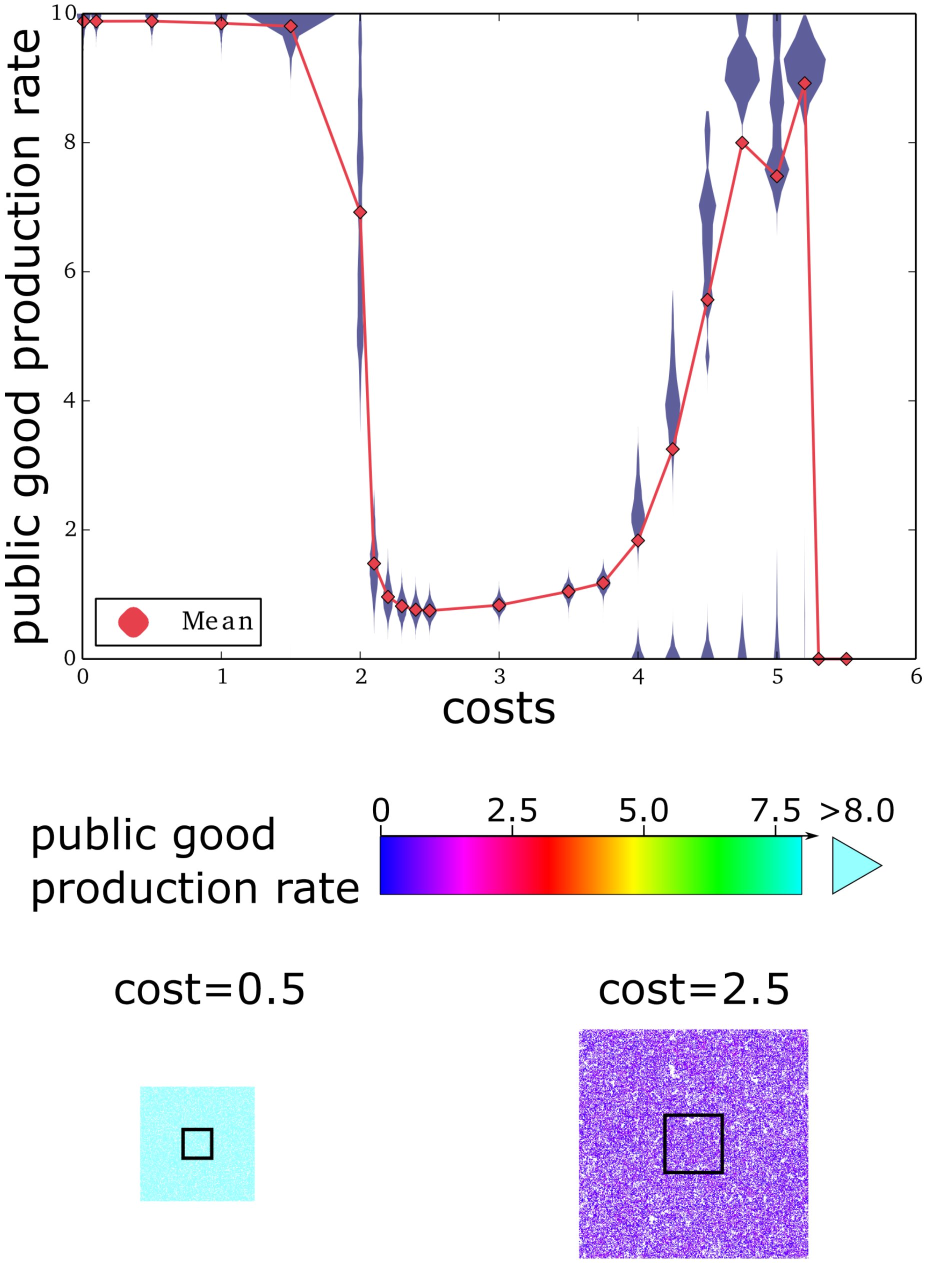

Figure S1: Evolutionary steady state of public good production for different costs (same figure as Fig.2). The snapshots depict the full lattice for $c = 0.5$ and $c = 2.5$. The black square marks the part of the snapshot that is diisplayed in Fig. 2.

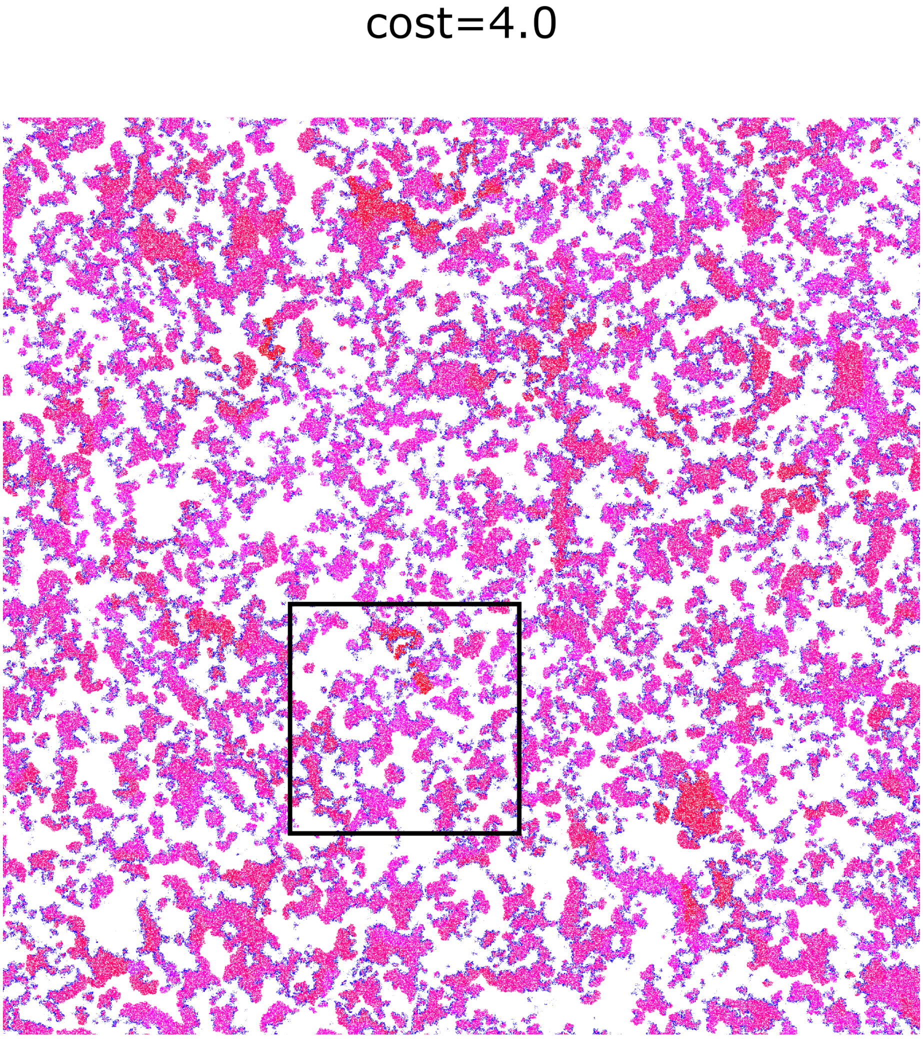

Figure S2: Snapshot of the full lattice for $c = 4.0$.The black square marks the part of the snapshot that is diisplayed in Fig. 2.

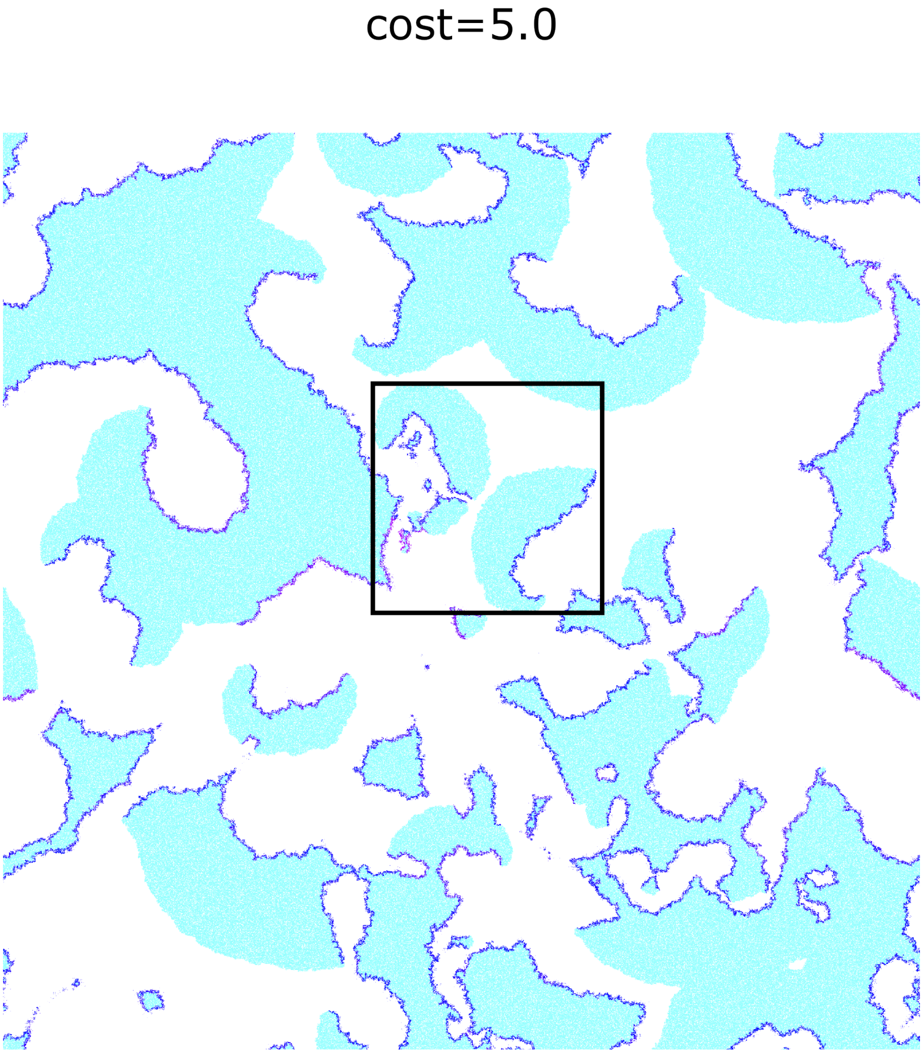

Figure S3: Snapshot of the full lattice for $c = 5.0$.The black square marks the part of the snapshot that is displayed in Fig. 2.

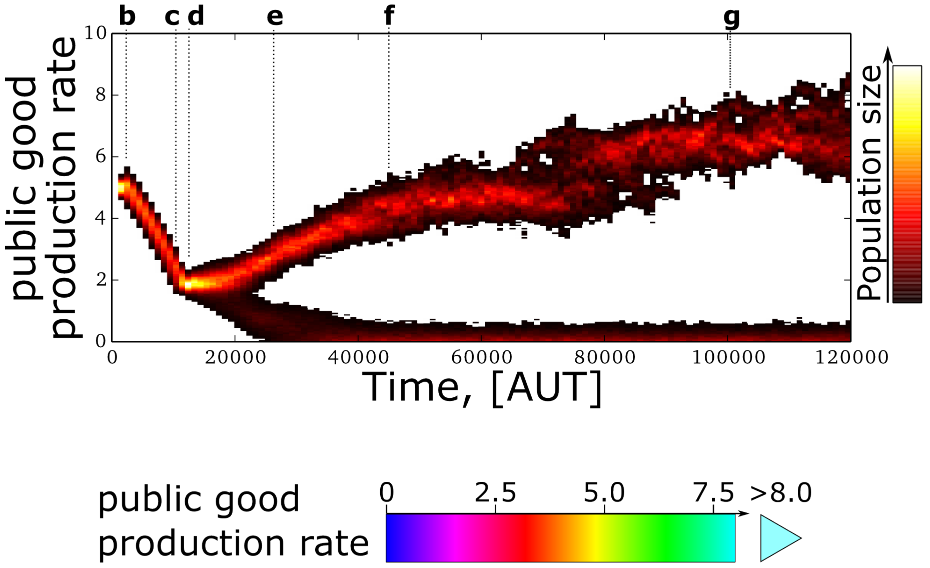

Figure S4: Evolutionary dynamics of public good production for $c = 4.5$ (same figure as Fig.3). This figure is meant for reference to the following.

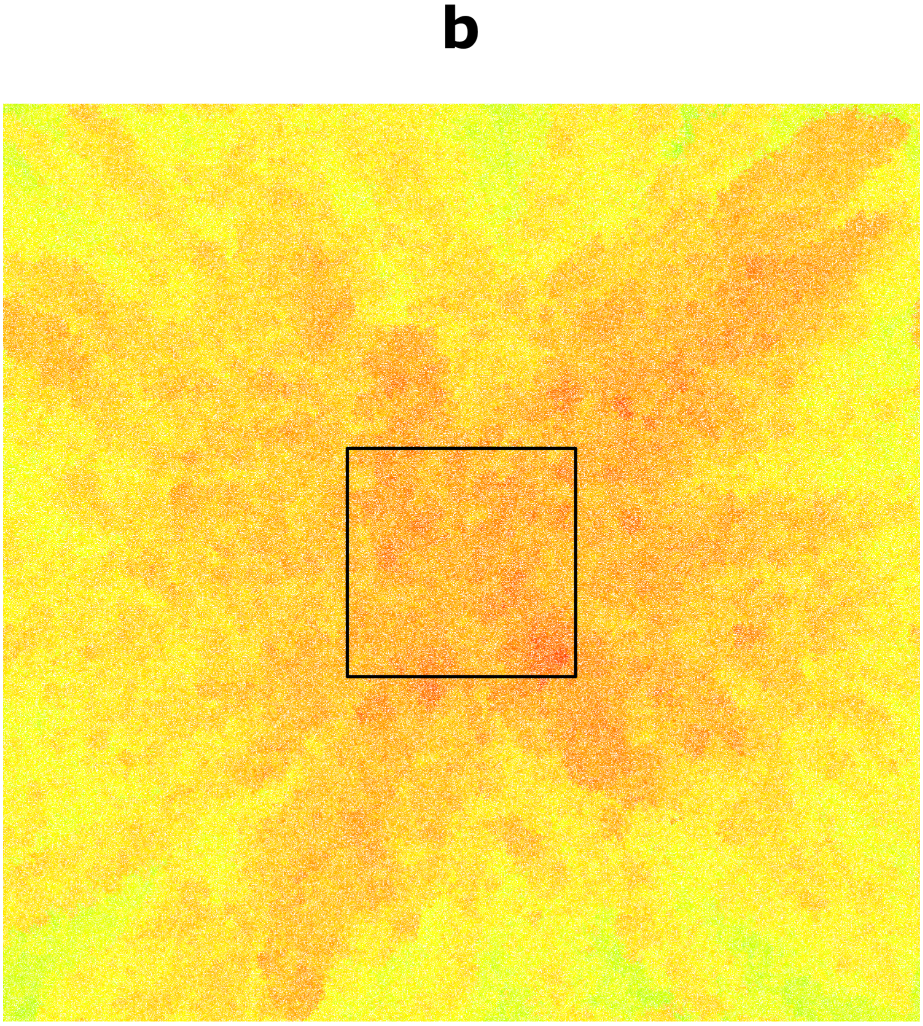

Figure S5: Snapshot of the full lattice at time point **b** of Fig. S4.

c

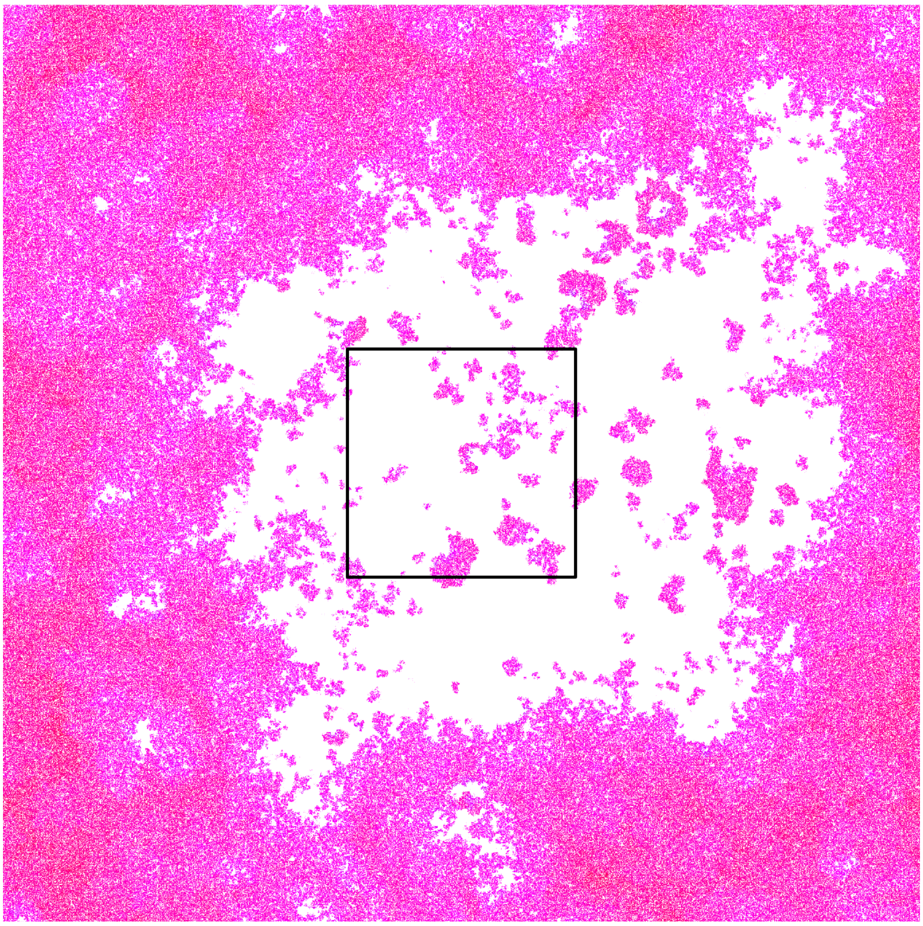

Figure S6: Snapshot of the full lattice at time point **c** of Fig. S4.

d

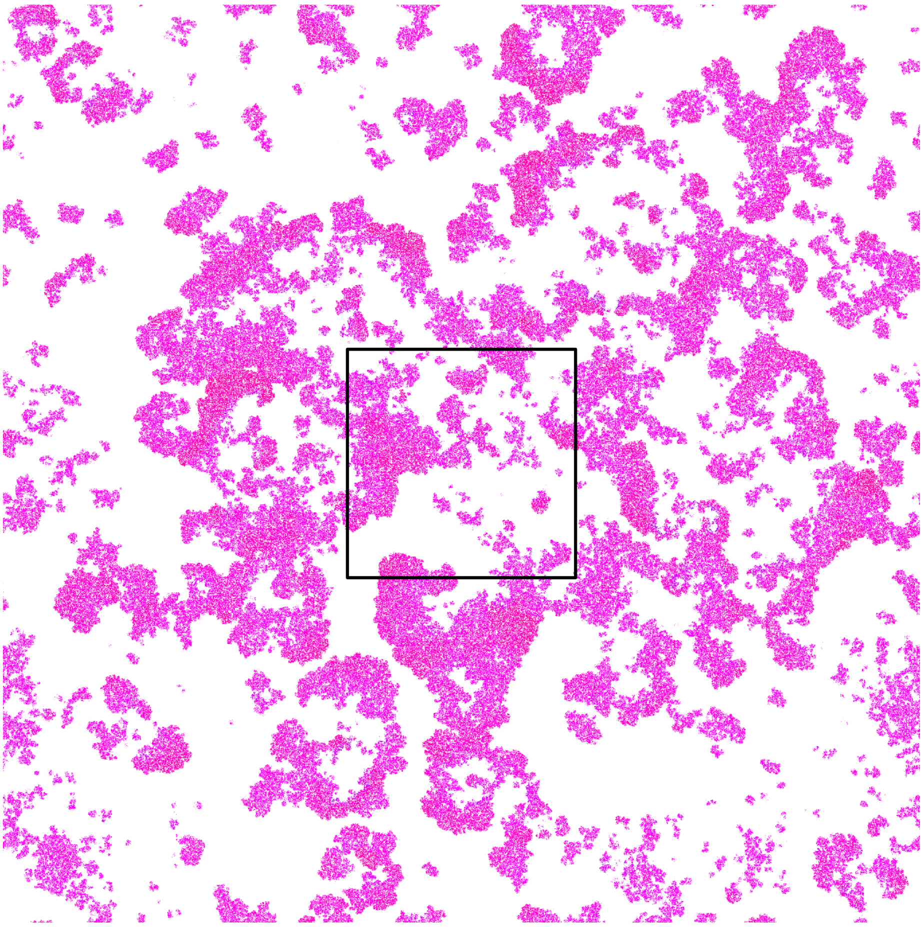

Figure S7: Snapshot of the full lattice at time point **d** of Fig. S4.

e

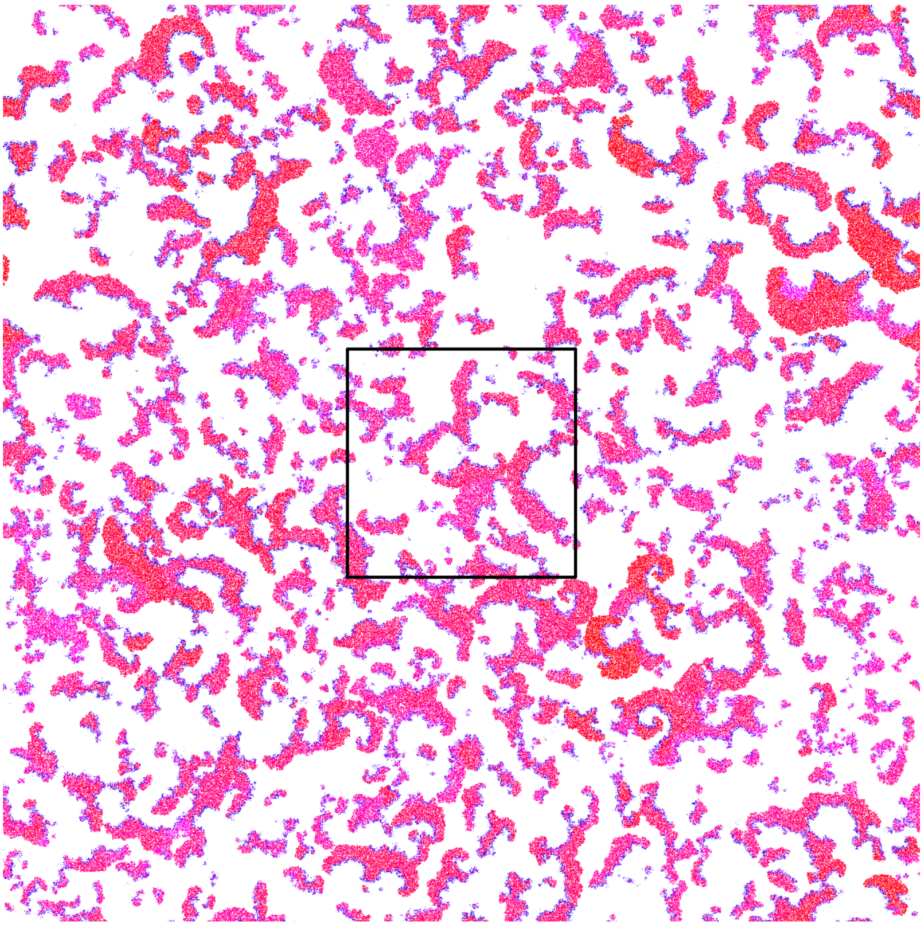

Figure S8: Snapshot of the full lattice at time point **e** of Fig. S4.

f

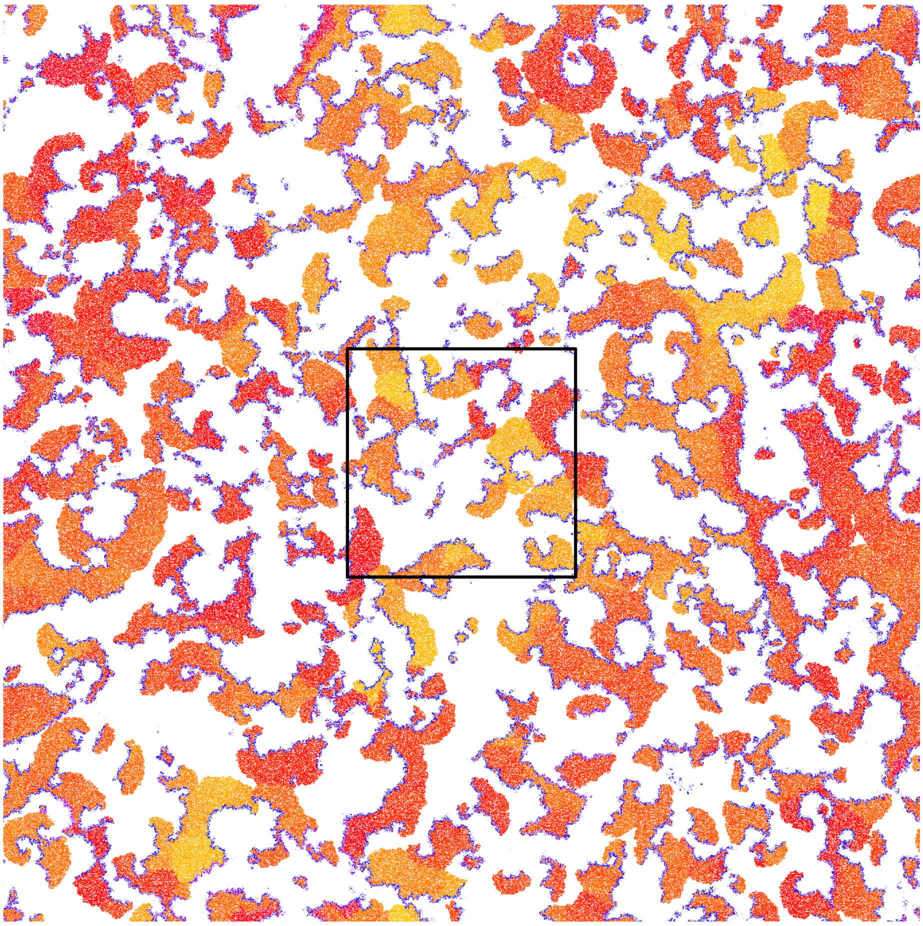

Figure S9: Snapshot of the full lattice at time point **f** of Fig. S4.

g

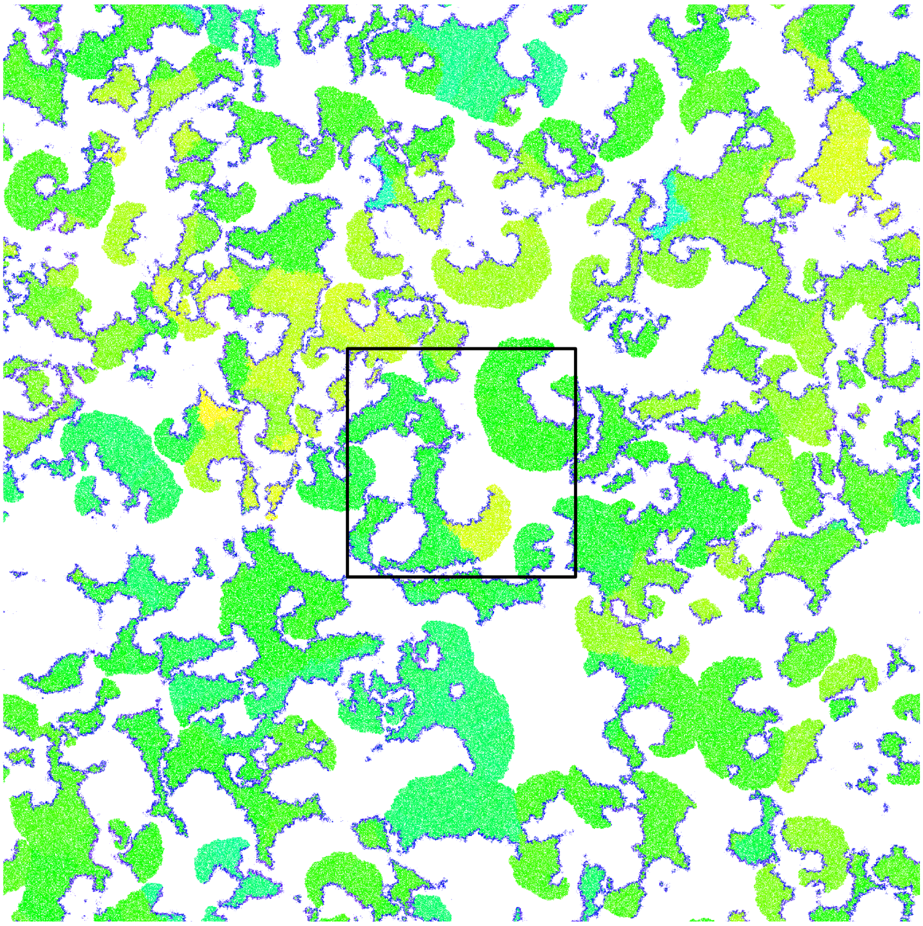

Figure S10: Snapshot of the full lattice at time point **g** of Fig. S4.

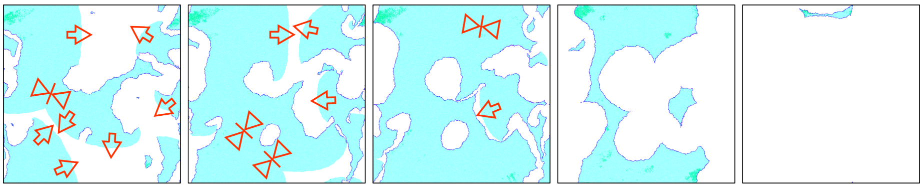

Figure S11: Giant wave-wave collisions may lead to extinction with high costs. Arrows indicate the direction of expansion of the waves, the ribbon-like symbols indicate where two wave-fronts have collided. Parameter. Costs = 5.3

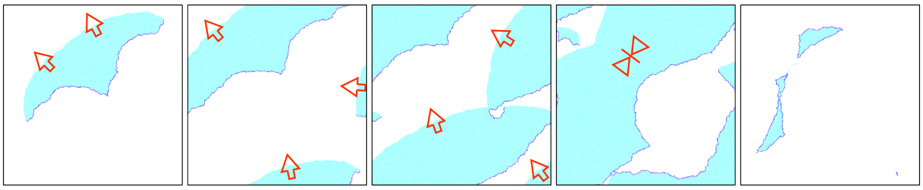

Figure S12: Giant single waves may touch their own back, letting selfish individuals invade the cooperators in the front. Under high costs this may lead to extinction. Arrows indicate the direction of expansion of the wave, the ribbon-like symbols indicate where two sides of the same wave-front collided on each other. Parameters. Costs=5.3

4 In a well-mixed system at high costs, only selfish behaviour is selected

In the main text, we have shown that cooperation increases for larger costs. The spatial organisation of the cooperative and the selfish lineage play a fundamental role in this. In Fig S13 we show that for high costs in well mixed conditions (where spatial patterns are destroyed), evolution favours selfishness. Public good production rapidly decreases in the system until it reaches values that are too small for survival. There, global extinction ensues.

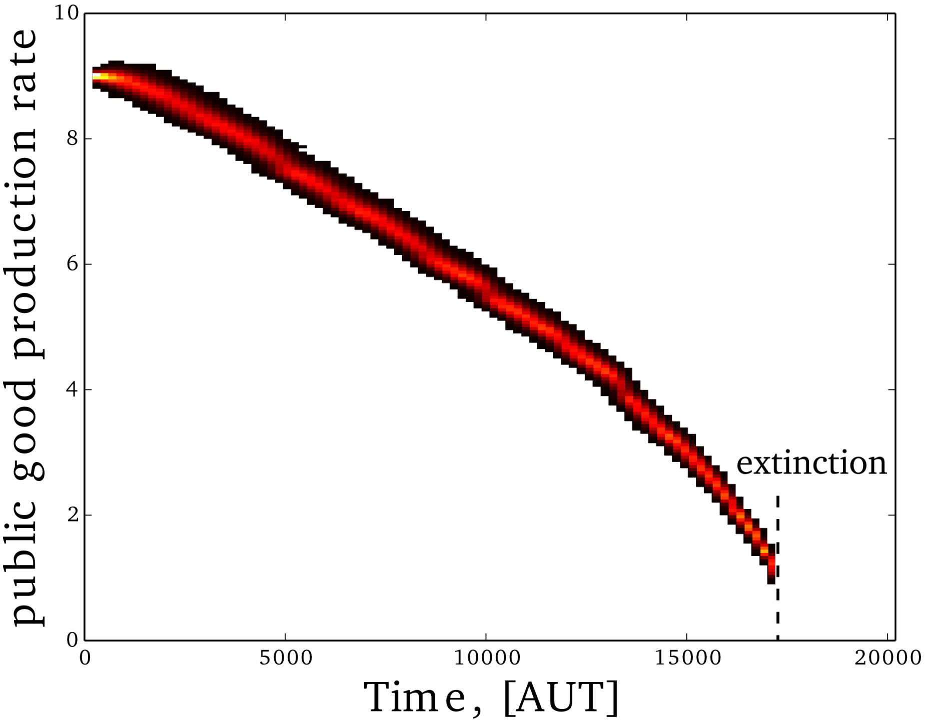

Figure S13: In a well mixed system with costs $c = 4.0$ cooperation is always minimised until extinction. Other parameters as in main text Fig. 3.

5 Consequences of removing cooperative or selfish individuals

We tested directly the interdependence between cooperative and selfish species by removing one species or the other from the system. In practice, we continued a simulation with high costs ($c = 4.5$) and after 10000 AUT we removed all individuals with public good production rate either larger or smaller than $p = 1.5$.

Removing all cooperators results in an almost instantaneous extinction of the selfish individuals (data not shown).

Removing all selfish individuals at one time point (Fig. S14a) results in a quick filling of the lattice with cooperators. With no selfish individuals to generate empty space, only individual-based selection for selfishness is present, and we observed a global decrease in public good production which leads to the same evolutionary dynamics observed in Fig. 3 of the main text. Eventually both the cooperative and the selfish species are restored and the system reaches the evolutionary state it had before removing all selfish individuals.

As a complementary approach, we ran a simulation and continuously removed selfish individuals, i.e. any individual with production rate $p < 1.5$ (costs are set to $c = 4.5$, as before). Removing selfish individuals does not allow for waves to form properly, which inhibits the evolutionary feedback between evolution and self-organisation described in the main text. Hence, we do not expect the evolution of large degrees of cooperation. This is confirmed by the results in Fig. S14b.

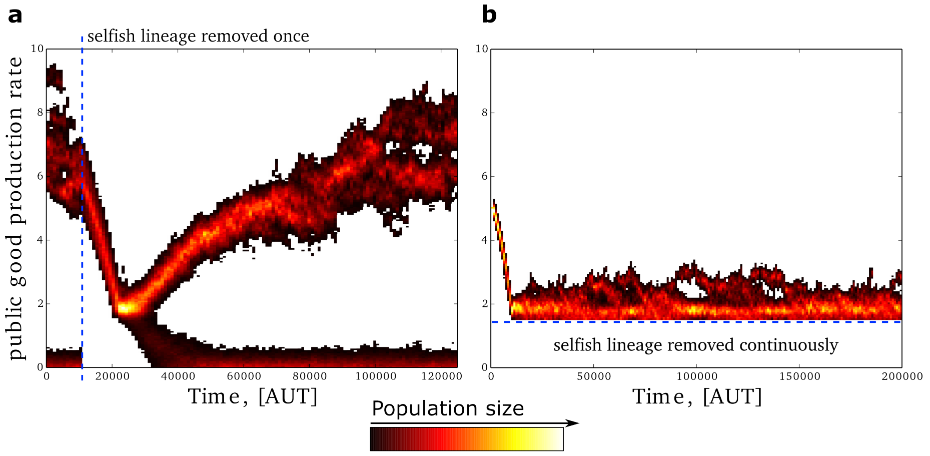

Figure S14: **a**: Removing all selfish individuals (at time 10000 AUT) results in a new evolutionary cycle which ultimately restores cooperative and selfish individuals. **b**: Continuously removing selfish individuals inhibits the evolution of cooperation.

6 Long term stability of the evolutionary steady state

The evolutionary dynamics of cooperators can be very noisy in the short run (Fig S15, upper pane). Mutliple lineages separate and persist long enough to evolve to different degrees of cooperation (public good production rates between 4 and 10). Nevertheless, we confirmed the long term stability of the steady state reached by the evolutionary dynamics by letting the system (shown in Main text Fig. 3) run much longer than the time scale needed to reach such steady state (Fig S15, lower pane).

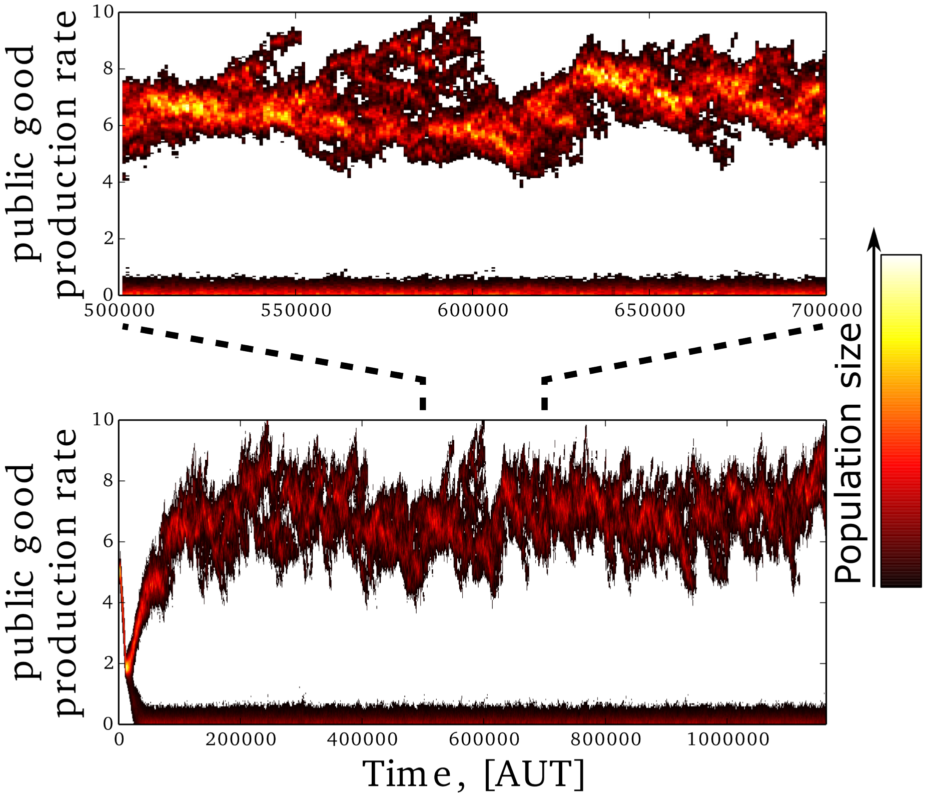

Figure S15: Short vs. long term evolutionary dynamics after reaching evolutionary steady state. The simulation is the same as in the Main text Fig. 3.

7 Moderate parameter changes do not affect results

7.1 *Death rate*

Individuals have an average life span of 1/[death rate] = 1/0.2 = 5 AUT. Decreasing the death rate to 0.1 does not change results (Fig. S16).

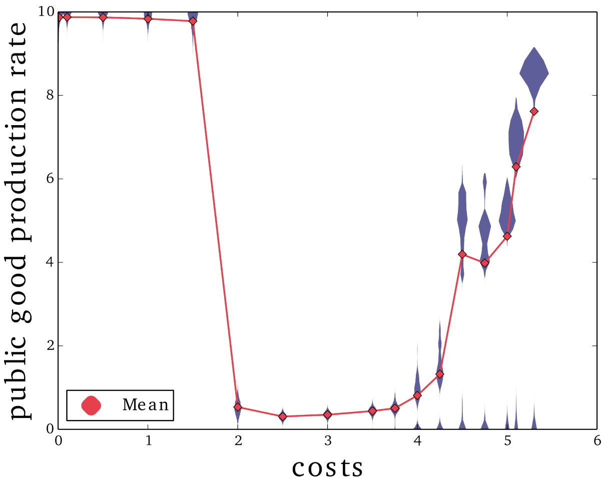

Figure S16: Final distribution for $k_{\mathrm{death}} = 0.1$. Other parameters (identical to main text Fig. 2) are b = 10, $k_{\mathrm{move}} = 0.02$, $\mu = 0.05$, $\delta = 0.1$

7.2 *Movement*

Movement is implemented as a random diffusive process (see Material and Methods in the main text). The probability that individuals move is kept to very low values ($p_{\mathrm{move}} = 0.02$ per individual, per time step) but not zero, so that spatial patterns do not get stuck in frozen configurations. This enhances the robustness of the observed spatial patterns. The stability of our results was checked by starting from backups and setting the rate of diffusion to 10 times higher (i.e. to $p_{\mathrm{move}} = 0.2$). We analysed three data points, corresponding to the three behaviours observed in the system for low, intermediate and high costs. Results are shown in Fig. S17, and are not qualitatively different. However, at higher costs ($c = 4.5$), we observe a much larger increase of public good production. A moderate increase in movement also increases the frequency with which other individuals are met. This constitutes an evolutionary advantage for selfish individuals, which translates in the generation of more empty space, thus giving cooperators a greater opportunity to expand into it.

The limit of very large diffusion is the well-mixed system. There, the advantage of selfish individuals becomes large, because spatial patterns cannot form. In this limit, cooperation breaks down at high costs, and for low costs with strong altruism (see Supplementary Section 9).

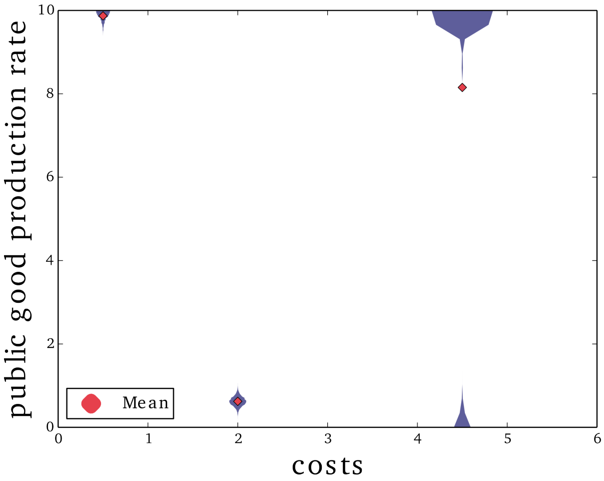

Figure S17: Final distribution for larger diffusion, $p_{\text{move}} = 0.2$. Other parameters are identical to main text Fig. 2

7.3 *Different values for benefits and costs*

In the main text, the benefits are kept always constant ($b = 10$) and simulations are run for different costs. Results do not change qualitatively if benefits are increased, as long as the benefit-to-cost ratio is kept approximately as in the main text. We report results for $b = 20$ in Fig. S18. For c = 10, the system shows large scale evolutionary fluctuations in public good production, with the selfish lineage occasionally producing more public good. However, the mechanism by which public good production increases remains the same as explained in the main text.

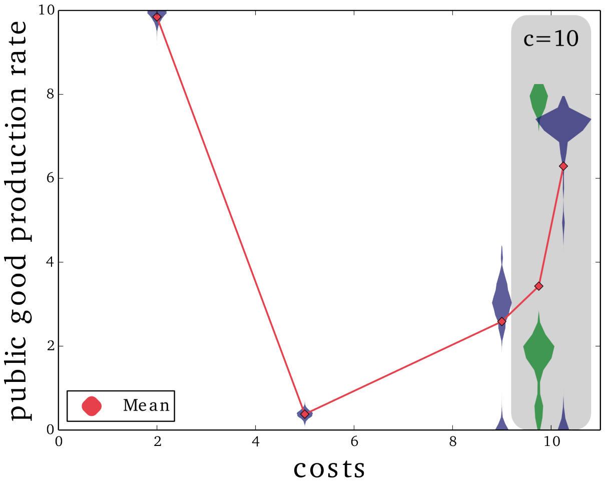

Figure S18: Distribution for benefits $b = 20$, twice as much as those used in the main text after reaching evolutionary steady state. Other parameters are the same as in main text Fig. 2. Both distributions in the shaded area correspond to c = 10 and are taken at different time points ($t_{\text{green}} = 450000$, $t_{\text{blue}} = 550000$ time steps).

8 Strong or weak altruism do not differ qualitatively

Whether an individual contributes positively to its own fitness or not is sometimes referred to as, respectively, weak and strong altruism [1, 2]. In our model, a fraction of public good remains by the individual that produced it. We can calculate the costs for which an individual has a positive fitness gain from its own public good production. The contribution to fitness an individual i gains from its own public good production is $f_{\text{self}} = \text{b}p_i/9 - \text{c}p_i$. For the benefits used in the main text, $\text{b} = 10$, f_{self} is positive only for $c \lesssim 1.1$. Thus we do not expect qualitative differences in the evolutionary dynamics for higher costs if we implemented strong altruism (where $f_{\text{self}} = -\text{c}p_i$, which can never be positive).

Fig.S19 confirms our prediction. Results are qualitatively the same to those described in the main text (Fig. 2) if public good is shared among neighbours, but no fraction is given to self. The maximum cost for which the system is viable is presumably higher than $c = 4.5$. However, spatial patterns become very large at higher costs, and an adequate lattice size render the simulation computationally infeasible (see Section 3 for a discussion on small lattice size and its consequences). More importantly, the evolutionary dynamics also lead to larger public good production with higher costs.

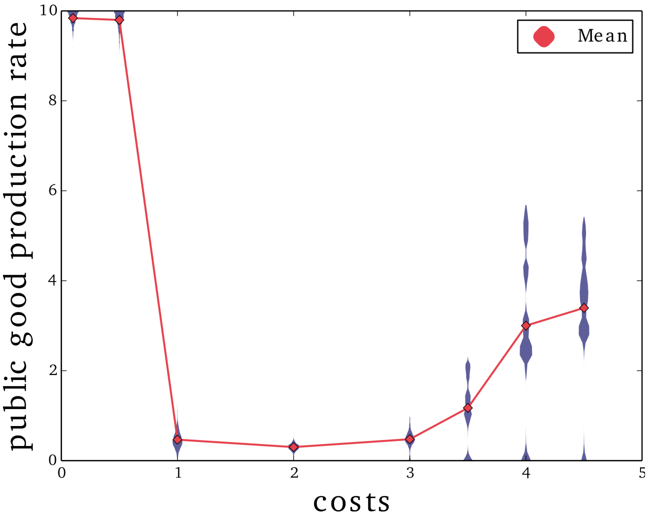

Figure S19: Increasing costs lead to increased cooperation when individuals do not retain the public good they produce. Parameters: $\text{b} = 10$, $k_{\text{death}} = 0.1$, $k_{\text{move}} = 0.02$, $\mu = 0.05$, $\delta = 0.1$

9 Under well-mixed conditions, strong or weak altruism differ at low costs

As shown in Section 8, the spatial system is largely indifferent to whether a fraction of produced public good is retained by the producer or not. Instead, if the spatial patterns are destroyed by well-mixing the system, we can observe that at lower costs, weak altruism leads to maximising public good production, while under strong altruism the system goes extinct (b = 10, c = 0.5 in Fig S20, left pane). When public good production costs are higher, the system goes extinct for both strong and weak altruism (b = 10, c = 4.0 in Fig S20, right pane).

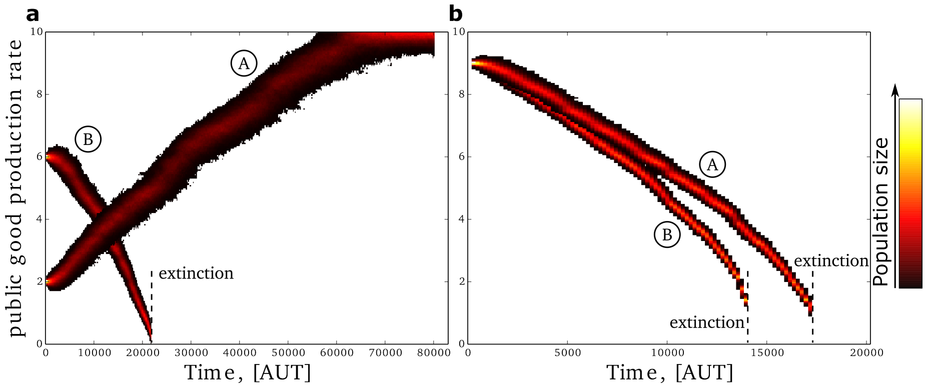

Figure S20: **a** With smaller costs $c = 0.5$, when individuals retain a fraction of public good they produce (A), public good production is maximised in the long run. In contrast, when producers do not retain a fraction of their own public good cooperation is minimised until extinction (B). **b** Whether individuals retain (A), or not (B) a fraction of public good they produce, in well mixed system with high costs $c = 4.0$ cooperation is always minimised until extinction. Notice that (A) is identical to Fig. S13.

References

[1] Wilson, D.S.: Structured demes and trait-group variation. American naturalist, 606–610 (1979)

[2] Fletcher, J.A., Doebeli, M.: A simple and general explanation for the evolution of altruism. Proceedings of the Royal Society B: Biological Sciences **276**(1654), 13–19 (2009)
